# Clinical characteristics and long-term prognosis of female patients with acute coronary syndrome

**DOI:** 10.3389/fcvm.2024.1447533

**Published:** 2024-08-23

**Authors:** Mar Rocamora-Horrach, Óscar M. Peiró, Alfredo Bardají, Javier Flores-Benítez, Miguel Ivorra-Cámara, Anna Carrasquer, José Luis Ferreiro

**Affiliations:** ^1^ Department of Cardiology, Joan XXIII University Hospital, Tarragona, Spain; ^2^ Pere Virgili Health Research Institute, Rovira i Virgili University, Tarragona, Spain; ^3^ Department of Medicine and Surgery, Rovira i Virgili University, Tarragona, Spain

**Keywords:** cardiovascular diseases, acute coronary syndrome, women’s health, prognosis, health equity

## Abstract

**Background:**

Cardiovascular disease has traditionally been studied predominantly in men, but understanding its manifestations in women is crucial for effective management. This study aims to evaluate the long-term prognosis of female patients with acute coronary syndrome (ACS) within a tertiary hospital setting in Spain.

**Methods:**

Retrospective observational study based on a cohort of consecutive hospitalized patients with ACS from January 2009 to December 2014. Data on demographics, risk factors, treatment, and outcomes were collected, with a median follow-up of 9.2 years.

**Results:**

Women with ACS, constituting 27.3% of 2,330 patients, were older and had a higher prevalence of cardiovascular risk factors such as obesity, hypertension, and diabetes mellitus compared to men. They presented with more non-ST-segment elevation myocardial infarction and underwent less coronary angiography. Female patients were also less likely to be treated with acetylsalicylic acid, a second antiplatelet drug, or statins. Despite initial higher mortality rates [hazard ratio (HR) 1.30; 95% confidence interval (CI) 1.13–1.49; *p* < 0.001], female patients exhibited a more favorable long-term prognosis after adjustments (adjusted HR 0.82; 95% CI 0.71–0.96; *p* = 0.014), even in the subgroup analysis excluding patients with unstable angina.

**Conclusions:**

Women with ACS are more comorbid, but after adjustments, female sex appears to be a protective factor that confers a better long-term prognosis.

## 1 Introduction

Cardiovascular disease (CVD) has traditionally been considered a male-centric health concern. Over the past five decades, CVD's age-adjusted mortality rates have decreased, although the decline has been less pronounced in women compared to men (1–3). Thus, comprehending the unique aspects of cardiovascular health in women is crucial for effective prevention, accurate diagnosis, appropriate treatment, and better prognosis (4, 5).

In several worldwide cohorts of patients with acute coronary syndrome (ACS), women are older and have more comorbidities than men (6, 7). They often exhibit a peculiar clinical presentation, leading to extended diagnostic intervals and delayed medical intervention (8). Women less frequently undergo diagnostic coronary angiography and are less prone to receive guideline-directed medical treatment (9–12). However, uncertainties concerning the long-term prognosis of ACS in women and its impacting factors exist. Some studies suggest a higher risk among women (13); others attribute the worse prognosis to higher comorbidity in women rather than female sex itself (14–16), while a few suggest female sex as a protective factor in ACS (17–19). Thus, the role that female sex plays in the prognosis of ACS remains undetermined.

Therefore, the aim of our study, conducted on a substantial cohort of consecutive patients with ACS in routine clinical practice at a tertiary hospital, is to evaluate the long-term prognostic impact of female sex in ACS.

## 2 Methods

### 2.1 Study design and patient selection

**Figure 1:**
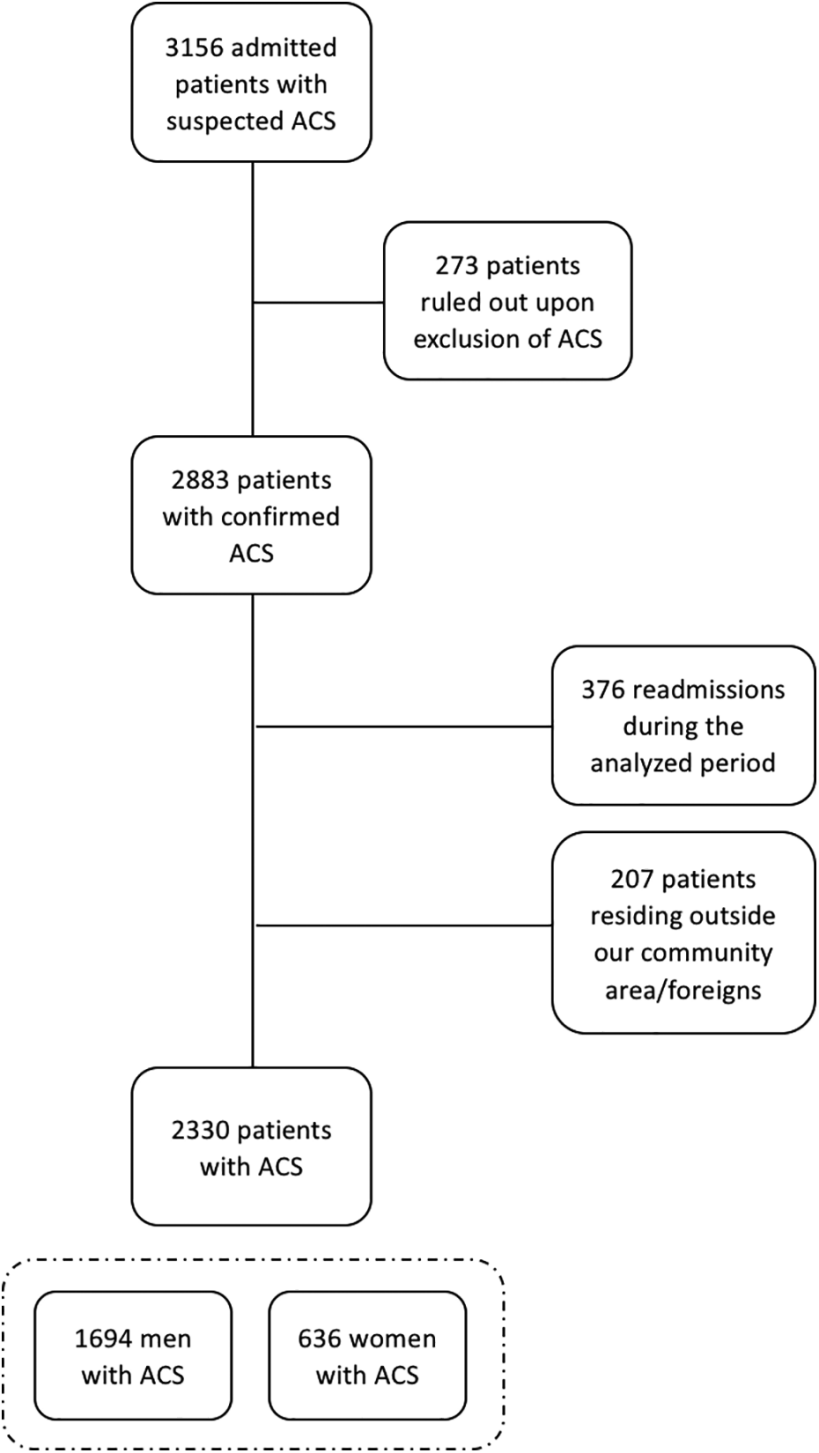
Flow chart of the study population including the initial number of patients evaluated and finally included in the study. ACS, acute coronary syndrome.

This retrospective observational study enrolled consecutive hospitalized patients with ACS at our tertiary hospital in Tarragona, Spain, from January 2009 to December 2014, and in which long-term follow-up could be ensured. Hence, patients lacking 1-year follow-up data from the index event were excluded, either because of being foreign or residing outside our reference area (Figure 1). The diagnosis of ST-segment elevation myocardial infarction (STEMI), non-ST-segment elevation myocardial infarction (NSTEMI), and unstable angina (UA) was established according to the criteria in the current European Guidelines at the time of the study (20, 21).

### 2.2 Data collection

Demographic information, key cardiovascular risk factors, and history of cardiovascular disease, as well as ongoing pharmacological treatment were collected when the patients were hospitalized for ACS. In addition, data regarding symptom experience during the index event, time in seeking medical care, first recorded vital signs, Killip classification at admission, initial EKG, key analytical indicators [including cardiac troponin I (Troponin I-Ultra, Advia Centaur, Siemens Healthineers, Erlanger, Germany) value and estimated glomerular filtration rate using CKD-EPI equation], and original GRACE score (22) were gathered. Furthermore, in-hospital pharmacological treatment (based on valid European Society of Cardiology guidelines at that time), left ventricular ejection fraction (LVEF) (distinguishing patients with LVEF <40%), reperfusion strategy [either by thrombolysis or primary percutaneous coronary intervention (PCI)], and coronary angiography results, if performed, were registered. All the previous data were extracted directly from medical records, including admission and discharge notes, as well as reports of complimentary test results.

### 2.3 Post-discharge follow-up and outcomes

Long-term follow-up, using regional electronic medical records, ensured ongoing monitoring despite residential changes within the same autonomous community. This enabled the acquisition of data on adverse events such as myocardial infarction (MI), all-cause death, cerebrovascular disease, major adverse cardiovascular events (MACE) composed of the latter three components, and hospitalization for heart failure (HF).

### 2.4 Statistical analysis

Categorical variables are expressed as numbers and percentages and comparisons were performed with chi-squared tests. Categorical data are expressed as the median and interquartile range and comparisons were done with the Mann–Whitney *U*-test. Survival probabilities were estimated by the Kaplan–Meier method and compared with the Log-rank test. To determine whether female sex was an independent predictor of cardiovascular events, univariable and multivariable Cox regressions were performed with the backward stepwise procedure, with inclusion and exclusion criteria set at 0.05 and 0.10, respectively. In the multivariable analysis, clinically relevant and significant variables in the univariable analysis were included; for this specific study, all variables were statistically significant at *p* <0.05. Therefore, multivariable Cox regression analysis was adjusted for age, current smoker, hypertension, diabetes mellitus, medical history of MI, medical history of HF, peripheral artery disease (PAD), estimated glomerular filtration rate at admission, LVEF <40%, discharge treatment with a second antiplatelet drug and statins. The proportional hazards assumption was analyzed by Schoenfeld residuals. Multicollinearity was searched by calculating the variance inflation factor. For MI, cerebrovascular disease and HF-related hospitalization during follow-up, all-cause death was included in all the analyses as a competing risk, and the Gray method was used. Differences were considered statistically significant at *p* < 0.05. STATA 14.2 (StataCorp, College Station, TX, USA) was used for statistical analysis.

### 2.5 Ethical considerations

This research was approved by the local ethics committee CEIM (December 2008) and adheres to the principles outlined in the Declaration of Helsinki. Owing to the retrospective nature of the gathered data and the absence of clinical intervention, we were exempted from the requirement to seek informed consent.

## 3 Results

### 3.1 Baseline characteristics

A total of 2,330 patients were included in the study, of which 636 (27.3%) were women. As summarized in Table 1, the women were older than the men and had a higher prevalence of cardiovascular risk factors such as obesity, hypertension, and diabetes mellitus. In contrast, among the men, there were more smokers and they more frequently had a history of MI and PAD. No significant differences were observed for dyslipidemia, prior PCI, prior cerebrovascular disease, and chronic kidney disease, although a history of HF was more common in women.

**Table 1:** Baseline characteristics and main clinical features at admission.

Variable	Overall (*N* = 2,330)	Men (*N* = 1,694)	Women (*N* = 636)	*p*-value
Baseline characteristics				
Age (years)	66.7 (56.4–77.0)	64.5 (54.5–74.9)	73.3 (62.6–80.7)	<0.001
Current smoker	743 (33.4)	630 (39.4)	113 (18.1)	<0.001
Hypertension	1,588 (68.8)	1,099 (65.5)	489 (77.9)	<0.001
Diabetes mellitus	788 (34.3)	520 (31.1)	268 (42.6)	<0.001
Hypercholesterolemia	1,291 (56.1)	927 (55.4)	364 (57.9)	0.289
Obesity (BMI ≥30 kg/m^2^)	448 (24.2)	313 (22.9)	135 (28.0)	0.024
Prior myocardial infarction	454 (19.5)	349 (20.6)	105 (16.5)	0.026
Prior heart failure	82 (3.5)	46 (2.7)	36 (5.7)	0.001
Cerebrovascular disease	161 (6.9)	113 (6.7)	48 (7.6)	0.460
Peripheral arterial disease	221 (9.5)	183 (10.8)	38 (6.0)	<0.001
Chronic kidney disease	204 (8.8)	155 (9.2)	49 (7.7)	0.271
Prior PCI	246 (10.6)	188 (11.1)	58 (9.1)	0.165
Prior ambulatory treatment				
Acetylsalicylic acid	767 (34.0)	543 (33.1)	224 (36.3)	0.148
Second antiplatelet drug	288 (12.8)	221 (13.5)	67 (10.9)	0.097
Oral anticoagulant	127 (5.6)	77 (4.7)	50 (8.1)	0.002
Beta blockers	568 (25.1)	410 (25.0)	158 (25.6)	0.755
ACE inhibitors or ARBs	992 (43.9)	682 (41.5)	310 (50.2)	<0.001
MRAs	73 (3.2)	42 (2.6)	31 (5.0)	0.003
Oral antidiabetic drugs	406 (18.0)	281 (17.1)	125 (20.3)	0.083
Insulin	255 (11.3)	154 (9.4)	101 (16.4)	<0.001
Statins	977 (43.3)	684 (41.7)	293 (47.5)	0.014
Clinical presentation				
Atrial fibrillation/flutter	179 (7.7)	113 (6.7)	66 (10.4)	0.003
Killip class >I	433 (18.7)	293 (17.4)	140 (22.1)	0.009
GRACE score	111 (88–136)	107 (87–132)	121 (96–145)	<0.001
LVEF <40%	516 (22.3)	387 (23.0)	129 (20.4)	0.181
Glycaemia (mg/dl)	130 (103–179)	128 (103–173)	136 (105–204)	0.002
eGFR (ml/min/1.73 m^2^)	80 (56–96)	83 (60–97)	69 (49–90)	<0.001
LDL cholesterol (mg/dl)	98 (78–121)	97 (78–120)	100 (77–125)	0.124
HDL cholesterol (mg/dl)	37 (31–44)	36 (30–42)	41 (33–49)	<0.001

BMI, body mass index; PCI, percutaneous coronary intervention; ACE, angiotensin-converting enzyme; ARBs, angiotensin II receptor blockers; MRAs, mineralocorticoid receptor antagonists; eGFR, estimated glomerular filtration rate; LDL, low-density lipoprotein; HDL, high-density lipoprotein; LVEF, left ventricle ejection fraction.

Data represent number (%) or median (interquartile range).

### 3.2 Clinical characteristics of acute coronary syndrome

At admission for an ACS, women presented more frequently with atrial fibrillation/flutter and worse Killip class than men (Table 1). Although women had a worse GRACE score, they were less likely than men to undergo coronary angiography (women 72.4% vs. men 83.4%; *p* < 0.001), and even when patients with UA were excluded, they also were less likely to undergo coronary angiography (women 73.2% vs. men 86.2%; *p* < 0.001) (Table 2). Among patients who underwent coronary angiography, women had a higher prevalence of non-significant coronary stenosis (women 21.7% vs. men 7.1%; *p* < 0.001) and, as a result, fewer PCI were performed (women 46.5% vs. men 64.5%; *p* < 0.001). There was a non-significant trend toward a lower prevalence of three-vessel disease in women (women 15.1% vs. men 17.6%; *p* = 0.227) (Table 2). During hospitalization, the prevalence of moderate/severe left ventricular systolic dysfunction (LVEF <40%) was similar between women and men, but women experienced higher mortality (women 6.5% vs. men 3.5%; *p* = 0.002).

**Table 2:** Early management of acute coronary syndrome by sex.

Variable	Overall (*N* = 2,330)	Men (*N* = 1,694)	Women (*N* = 636)	*p*-value
Coronary angiography				
Total procedures	1,873 (80.4)	1,413 (83.4)	460 (72.4)	<0.001
Non-significant coronary stenosis^a^	191 (10.7)	95 (7.1)	96 (21.7)	<0.001
Significant three vessels stenosis^a^	303 (17.0)	236 (17.6)	67 (15.1)	0.227
PCI^a^	1,389 (59.6)	1,093 (64.5)	296 (46.5)	<0.001
In-hospital complications				
Death during hospitalization	101 (4.3)	60 (3.5)	41 (6.5)	0.002
Discharge diagnosis				
STEMI	931 (40.0)	722 (42.6)	209 (32.9)	<0.001
NSTEMI	1,082 (46.4)	756 (44.6)	326 (51.3)	0.004
UA	317 (13.6)	216 (12.8)	101 (15.9)	0.050
Discharge treatment				
Acetylsalicylic acid	2,009 (87.9)	1,494 (89.8)	515 (82.7)	<0.001
Second antiplatelet drug	1,720 (75.3)	1,314 (79.1)	406 (65.1)	<0.001
Oral anticoagulant	197 (8.6)	134 (8.1)	63 (10.1)	0.126
Beta blockers	1,553 (68.0)	1,158 (69.7)	395 (63.3)	0.003
ACE inhibitors or ARBs	1,362 (59.6)	989 (59.5)	373 (59.8)	0.907
MRAs	124 (5.4)	96 (5.8)	28 (4.5)	0.223
Oral antidiabetic drugs	422 (18.5)	278 (16.8)	144 (23.1)	0.001
Insulin	302 (13.2)	192 (11.6)	110 (17.6)	<0.001
Statins	1,957 (85.7)	1,453 (87.5)	504 (80.9)	<0.001

PCI, percutaneous coronary intervention; ACE, angiotensin-converting enzyme; ARBs, angiotensin II receptor blockers; MRAs, mineralocorticoid receptor antagonists; STEMI, ST-elevation myocardial infarction; NSTEMI, non-ST-elevation myocardial infarction; UA, unstable angina.

Data represent number (%) or median (interquartile range).

^a^ Data regarding patients who underwent coronary angiography.

As a discharge diagnosis, men had significantly more STEMI, but women had more NSTEMI and there was a trend toward more UA. Regarding discharge treatment, women were less likely to be treated with acetylsalicylic acid (women 82.7% vs. men 89.8%; *p* < 0.001) and with a second antiplatelet drug in dual antiplatelet therapy (women 65.1% vs. men 79.1%; *p* < 0.001), and this difference was still significant when we excluded those patients with UA for both treatments [acetylsalicylic acid (women 83.3% vs. men 89.8%; *p* < 0.001); second antiplatelet drug (women 67.7% vs. men 81.7%; *p* < 0.001)]; however, no difference was observed if we focused only on patients who underwent PCI (Table 2). Similarly, statins were prescribed less to women than men (women 80.9% vs. men 87.5%; *p* < 0.001), even when we excluded patients with UA (women 79.1% vs. men 86.8%; *p* < 0.001), and there was a non-significant trend toward less statin therapy when selecting only patients with significant coronary stenosis (women 87.1% vs. men 89.7%; *p* = 0.127) (Table 2).

### 3.3 Long-term prognosis

**Figure 2:**
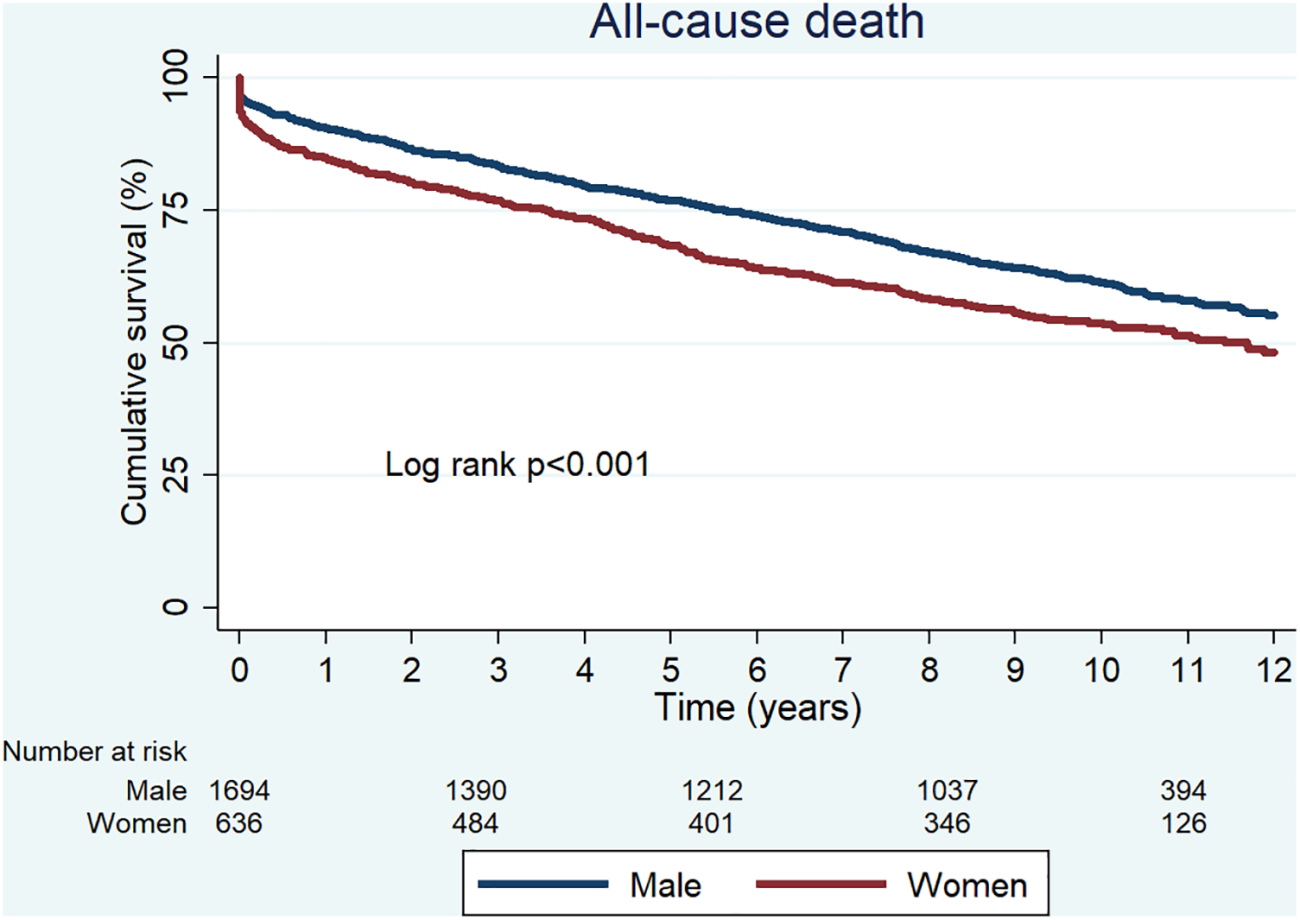
Unadjusted Kaplan–Meier curves for all-cause death.

All patients included in the study were successfully followed up beyond the 1-year mark. During a median follow-up of 9.2 years [interquartile range (IQR) 4.0–10.7], 963 patients had died. Among them, 665 (39.3%) were men and 298 (46.9%) were women. In an unadjusted analysis, women were at higher risk of all-cause death than men [unadjusted hazard ratio (HR) 1.30; 95% confidence interval (CI) 1.13–1.49; *p* < 0.001], but after adjustment, women were associated with lower risk of mortality (adjusted HR 0.82; 95% CI 0.71–0.96; *p* = 0.014) (Figure 2) (Tables 3, 4).

**Table 3:** Long-term risk of cardiovascular events in women versus men after an acute coronary syndrome.

	Women events (%)	Men events (%)	Women vs. men	
			Unadjusted HR (95% CI); *p*-value	Adjusted HR (95% CI); *p*-value
All-cause death	298 (46.9)	665 (39.3)	1.30 (1.13–1.49); *p* < 0.001	0.82 (0.71–0.96); *p* = 0.014
MACE	344 (54.1)	832 (49.1)	1.17 (1.04–1.33); *p* = 0.012	0.85 (0.74–0.97); *p* = 0.018
Myocardial infarction	82 (12.9)	256 (15.1)	0.84 (0.65–1.07); *p* = 0.164	Non-significant
Cerebrovascular disease	39 (6.1)	102 (6.0)	1.00 (0.69–1.44); *p* = 0.998	Non-significant
Heart failure	92 (14.5)	189 (11.2)	1.33 (1.04–1.71); *p* = 0.024	Non-significant

HR, hazard ratio; CI, confidence interval; MACE, major adverse cardiovascular events (all-cause death, myocardial infarction, and cerebrovascular disease).

Cardiovascular events including unadjusted and adjusted risk. Adjusted model include age, current smoker, hypertension, diabetes mellitus, medical history of MI, medical history of HF, PAD, estimated glomerular filtration rate at admission, LVEF <40%, discharge treatment with a second antiplatelet drug and statins.

**Table 4:** Long-term risk of all-cause death after an acute coronary syndrome.

Variables	Univariate cox regression		Multivariate cox regression	
	HR (95% CI)	*p*-value	HR (95% CI)	*p*-value
Age	1.09 (1.08–1.09)	<0.001	1.07 (1.06–1.08)	<0.001
Current smoker	1.42 (1.35–1.49)	<0.001	1.34 (1.11–1.62)	0.002
Hypertension	2.54 (2.15–3.01)	<0.001	—	—
Diabetes mellitus	2.18 (1.92–2.48)	<0.001	1.63 (1.42–1.87)	<0.001
Previous myocardial infarction	2.11 (1.84–2.43)	<0.001	1.32 (1.13–1.54)	<0.001
Previous heart failure	3.88 (3.03–4.97)	<0.001	1.56 (1.19–2.04)	0.001
Previous peripheral artery disease	2.65 (2.23–3.14)	<0.001	1.52 (1.25–1.84)	<0.001
eGFR at admission	1.03 (1.02–1.03)	<0.001	1.01 (1.01–1.02)	<0.001
LVEF <40%	2.41 (2.10–2.76)	<0.001	1.60 (1.38–1.87)	<0.001
Second antiplatelet drug^a^	0.52 (0.46–0.59)	<0.001	0.78 (0.68–0.90)	0.001
Statins^a^	0.41 (0.35–0.38)	<0.001	0.60 (0.51–0.72)	<0.001
Women	1.30 (1.13–1.49)	<0.001	0.82 (0.71–0.96)	0.014

HR indicates hazard ratio; CI, confidence interval; eGFR, estimated glomerular filtration rate; LVEF, left ventricle ejection fraction.

Univariate and multivariate Cox regression analysis.

^a^ Discharge treatment.

**Figure 3:**
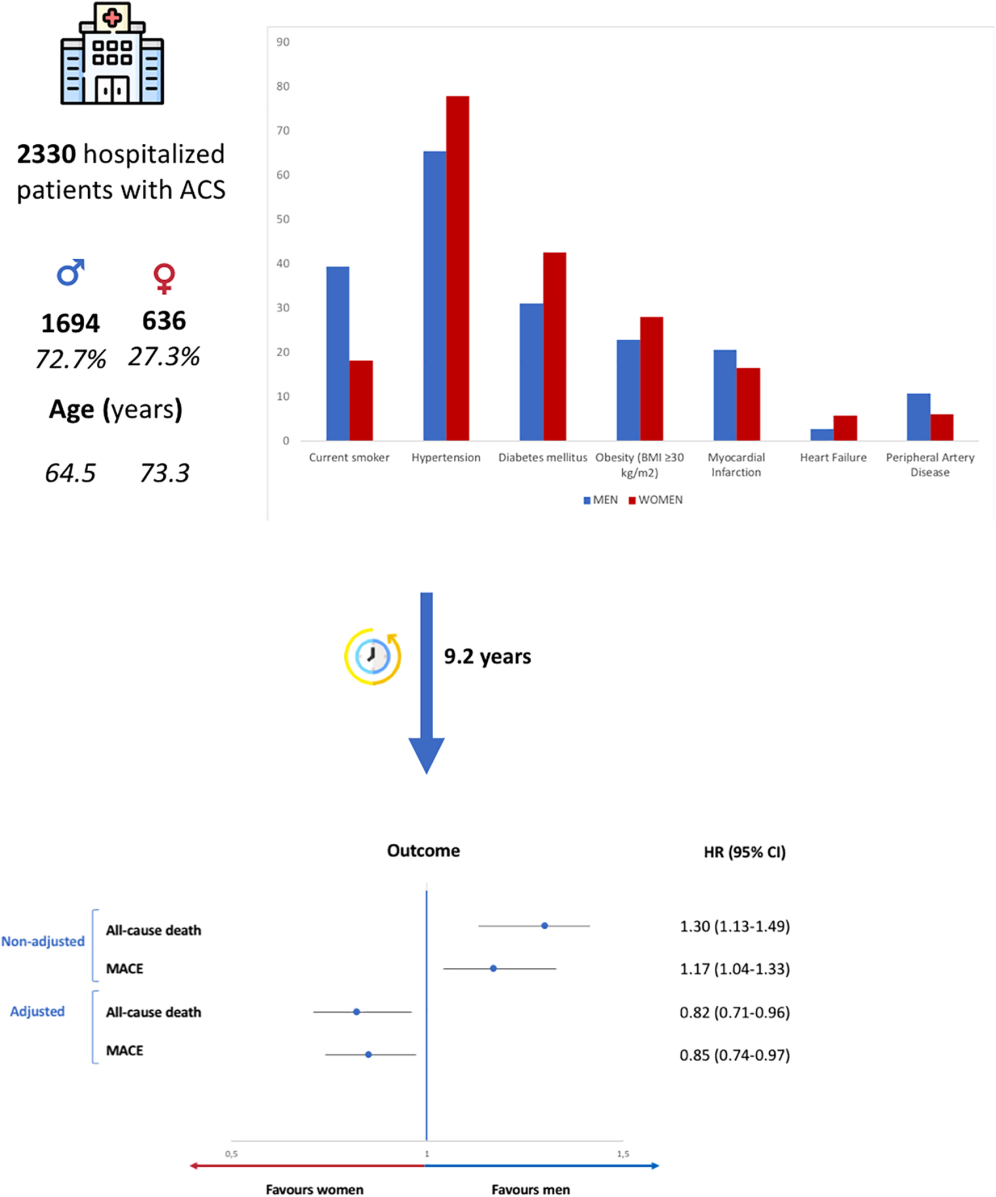
Summary of the design and main results of the study. ACS, acute coronary syndrome; MACE, major adverse cardiovascular events (all-cause death, myocardial infarction, and cerebrovascular disease). HR, hazard ratio.

A composite of MACE and individual cardiovascular events of MI, cerebrovascular disease, and HF were studied during follow-up. In an unadjusted analysis, women had an increased risk of MACE (unadjusted HR 1.17; 95% CI 1.04–1.33; *p* = 0.012) and HF (unadjusted HR 1.17; 95% CI 1.04–1.33; *p* = 0.012) than men but had a similar risk of MI (unadjusted HR 1.17; 95% CI 1.04–1.33; *p* = 0.012) and cerebrovascular disease (unadjusted HR 1.17; 95% CI 1.04–1.33; *p* = 0.012). However, after multivariate analysis, women were associated with a lower risk of MACE than men (adjusted HR 0.85; 95% CI 0.74–0.97; *p* = 0.018) (Central Illustration, Figure 3). The adjusted risk of MI, cerebrovascular disease, and HF did not differ between women and men.

In a subgroup analysis performed after excluding UA patients, women initially showed a higher risk of all-cause death (unadjusted HR 1.45; 95% CI 1.25–1.67; *p* < 0.001) and MACE (unadjusted HR 1.27; 95% CI 1.11–1.46; *p* < 0.001) than men in an unadjusted analysis. However, after multivariate analysis, the risk became equal between both sexes (Supplementary Table S1). In addition, both unadjusted and adjusted risk of long-term MI, cerebrovascular disease, and HF did not differ between women and men.

In NSTEMI patients, women were associated with a higher risk of all-cause death (unadjusted HR 1.34; 95% CI 1.12–1.60; *p* = 0.001) and MACE (unadjusted HR 1.23; 95% CI 1.04–1.45; *p* = 0.015) than men in an unadjusted analysis, but after multivariate analysis, the risk was the same in both sexes. Similarly, in STEMI patients, women had a higher unadjusted risk of all-cause death (unadjusted HR 1.46; 95% CI 1.14–1.88; *p* = 0.003) than men, which also equalized after multivariate analysis. In both NSTEMI and STEMI patients, the risk of long-term MI, cerebrovascular disease, and HF did not differ between sexes in either unadjusted or adjusted analyses nor did the risk of MACE in the latter.

## 4 Discussion

Despite greater comorbidity in women with ACS and a worse long-term crude prognosis, the latter was reversed after adjusting for other factors, thus female patients present a better long-term prognosis than their male counterparts.

From the surge of publications on ACS and sex in the 1990s, this topic has gained momentum within the scientific community. Sex-based disparities in CVD presentation, treatment, and mortality rates are well-documented, yet many questions remain unanswered regarding the underlying mechanisms (1, 4, 23).

Our study, in line with the conclusions of Sarma et al. (18), even considering patients with STEMI in addition to NSTEMI and encompassing a longer follow-up, underscores a higher comorbidity burden in women with ACS, as well as the evident difference in therapeutic strategies based on sex, greater prevalence of non-obstructive coronary artery disease (CAD) in women, and a better long-term prognosis of women with ACS compared to men.

In Spain, several studies have also investigated sex differences in ACS presentation, management, and prognostic implications for female patients (24–27). Our profile of patients with ACS and findings are consistent with those reported by García-García et al. (28), reflecting a reliable representation of the profile of our cohort of patients with ACS at the national level and providing external validation of our results. Also, we demonstrated that sex disparities in management persisted even after excluding patients diagnosed with UA.

Our Italian neighbors from Southern Europe describe a female ACS profile similar to ours. D’Ascenzo et al. (29) conclude a higher in-hospital mortality regardless of age, without examining prognosis after hospital discharge. Meanwhile, De Luca et al. (30) observe a similar distribution of CAD and long-term prognosis between sexes, with this observation limited to patients with STEMI.

Considering the scenario of ACS in Central Europe, and congruent with our findings, Koek et al. (31) highlight an unfavorable crude prognosis for women both in the short and long term, that is reversed after adjusting for age. In contrast, Sörensen et al. (32) report a similar 2-year prognosis between both sexes, with men exhibiting more frequent revascularization and rehospitalization. Similarly, women present fewer obstructive CAD, aligning with previous observations.

Furthermore, if we consider the situation in Northern Europe, adverse events following an MI have significantly reduced over the years and, apparently, the prognosis of patients with ACS for up to 5 years is comparable between sexes (33–35).

Finally, in harmony with those studies showing a lower burden of CAD in women (18, 28), the adjusted long-term prognosis favors female patients. Conversely, in studies where the CAD profile is similar between sexes (29, 30), the adjusted long-term prognosis does not differ.

Therefore, our study provides valuable confirmation and reinforcement of previous larger-scale trials’ findings regarding a longer-term prognosis of women with ACS. It strengthens the evidence by suggesting that their prognosis is not only not worse than men's but actually better, which could be attributed to a lower prevalence of obstructed CAD in women.

### 4.1 Study limitations

This study is subject to several limitations. It is a unicentric retrospective observational study and, as such, provides only associative evidence. Also, we do not have data on cause-specific mortality, yet it has been described that non-cardiovascular mortality represents a non-negligible percentage of long-term deaths in patients with ACS (36). Finally, treatment after hospital discharge and throughout the study period was not collected; hence, the impact of post-discharge treatment on outcomes cannot be assessed, potentially limiting the generalizability of the findings. These limitations highlight the need for better data collection on ACS in Spain, requiring increased efforts to register cases and standardize patient evaluation criteria.

## 5 Conclusions

While initially showing higher risks of mortality and MACE, women's prognosis appears to improve after adjusting for other covariables, likely due to a more favorable CAD profile compared to men, and possibly indicating a protective effect of female sex.

## Data Availability

The raw data supporting the conclusions of this article will be made available by the authors, without undue reservation.
