# Supplementary material for: Clinical characteristics and long-term prognosis of female patients with acute coronary syndrome
Source: Front Cardiovasc Med. 2024 Aug 23;11:1447533. doi: 10.3389/fcvm.2024.1447533 (PMC11377307; doi:10.3389/fcvm.2024.1447533)
Supplement: Supplementary file 1 [file Table1.docx]

**Supplementary table 1. Long-term risk of all-cause death after an acute myocardial infarction. Univariate and multivariate Cox regression analysis.**

|  | **Univariate Cox Regression** | | **Multivariate Cox Regression** | |
| --- | --- | --- | --- | --- |
| **Variables** | **HR (95% CI)** | **P-Value** | **HR (95% CI)** | **P-Value** |
| Age | 1.09 (1.08-1.09) | <0.001 | 1.07 (1.06-1.08) | <0.001 |
| Current smoker | 1.40 (1.34-1.47) | <0.001 | 1.44 (1.17-1.77) | <0.001 |
| Hypertension | 2.68 (2.25-3.20) | <0.001 | - | - |
| Diabetes mellitus | 2.22 (1.94-2.55) | <0.001 | 1.60 (1.38-1.86) | <0.001 |
| Previous myocardial infarction | 2.31 (1.99-2.68) | <0.001 | 1.48 (1.25-1.74) | <0.001 |
| Previous heart failure | 4.17 (3.22-5.42) | <0.001 | 1.79 (1.35-2.37) | <0.001 |
| Previous peripheral artery disease | 2.60 (2.17-3.12) | <0.001 | 1.56 (1.28-1.90) | <0.001 |
| eGFR at admission | 1.03 (1.02-1.03) | <0.001 | 1.01 (1.01-1.02) | <0.001 |
| LVEF<40% | 2.43 (2.11-2.80) | <0.001 | 1.56 (1.33-1.82) | <0.001 |
| Second antiplatelet drug* | 0.43 (0.38-0.49) | <0.001 | 0.73 (0.62-0.85) | <0.001 |
| Statins* | 0.39 (0.33-0.46) | <0.001 | 0.64 (0.53-0.77) | <0.001 |
| **Women** | **1.45 (1.25-1.67)** | **<0.001** | **-** | **-** |

*Discharge treatment.

HR indicates hazard ratio; CI: confidence interval; eGFR: estimated glomerular filtration rate; LVEF: left ventricle ejection fraction.
